# *Rubuspuyumaensis*, a new species of Rosaceae from Taiwan

**DOI:** 10.3897/BDJ.12.e115431

**Published:** 2024-01-25

**Authors:** Po-Hao Chen, Juinn-Yih Huang, An-Ching Chung

**Affiliations:** 1 Liouguei Research Center, Taiwan Forest Research Institute, Kaohsiung, Taiwan Liouguei Research Center, Taiwan Forest Research Institute Kaohsiung Taiwan; 2 Graduate Institute of Bioresources, National Pingtung University of Science and Technology, Pingtung, Taiwan Graduate Institute of Bioresources, National Pingtung University of Science and Technology Pingtung Taiwan; 3 Independent researcher, Taipei, Taiwan Independent researcher Taipei Taiwan

**Keywords:** new taxon, Puyuma, *Rubushowii*, *R.refractus*, Taiwan

## Abstract

**Background:**

The genus *Rubus* L. (Rosaceae), comprising approximately 750 species and classified into 12 subgenera, is distributed worldwide and is one of the largest plant genera. In Taiwan, *Rubus* comprises 41 taxa, including 35 species, three varieties and three hybrids. Amongst the genus *Rubus*, the species, previously recorded as *R.howii* in Taiwan, was misidentified and this study recognised it as a new species.

**New information:**

Due to its distribution mainly in south-eastern Taiwan, we named this new species as *Rubuspuyumaensis*, after the local aborigine tribe Puyuma. Taxonomic descriptions and colour photographs of the new species are provided to assist in identification. *R.puyumaensis* is most similar to *R.howii* and *R.refractus*. They can be distinguished by the colour of young leaves, leaf shape, arrangement of florets, trichomes of inflorescences, size of sepal lobes, petal colour, types and trichomes of filaments and the length of stamens and pistils.

## Introduction

The genus *Rubus* L. (Rosaceae), comprising approximately 863 species (Huang et al. 2023) and classified into 12 subgenera, is distributed worldwide and is one of the largest plant genera (Romoleroux 1996, Alice and Campbell 1999, Lu and Boufford 2003). It originated in North America and migrated to the other continents, hence the high diversity in the world, especially in Asia and Europe (Carter et al. 2019), with only a few species being discovered in tropical areas and in the Southern Hemisphere (Kalkman 2004).

In Taiwan, *Rubus* comprises 41 taxa, including 35 species, three varieties and three hybrids (Huang and Hu 2009, Chung et al. 2017), amongst which *Rubushowii* Merr. & Chun was reported endemic to Hainan Island, China (Lu and Boufford 2003, Chen et al. 2016) and, further afield, newly recorded in Taiwan (Lu and Yang 1980), with the follow-up revision of *Rubus* in Taiwan using *R.howii* as the scientific name of this species (Huang and Hu 2009). In recent years, the second author of this study found numerous populations of this species in Taitung, Taiwan and noted the flowers and fruits. After comparing this species in detail with the type (*F.C. How 72000*, A), reviewing the specimens of *R.howii* placed in other herbaria and, at same time, viewing the relevant study papers (Lu and Boufford 2003, Pelser et al. 2011, Gupta and Dash 2018, Welch 2021, Espinel-Ortiz and Romoleroux 2021, Huang et al. 2022), we recognised that the species previously recorded as *R.howii* in Taiwan were misidentified. We then confirmed it as a new species.

## Materials and methods

We compared the specimens of *Rubushowii* in Herbaria IBK, IBSC and PE to the species recorded as *R.howii* in Taiwan and confirmed it as a new species that had not been named. After measuring and describing the characteristics of the population at Chinshui Farm and Lichia logging tract, we collected the specimens and deposited them in the Herbarium HAST. We also examined the specimens of this species in other herbaria in Taiwan, HAST, PPI, TAI, TAIE, TAIF, TCF and TNM to confirm the populations in the field.

## Taxon treatments

### Rubus puyumaensis

J.Y. Huang, P.H. Chen & A.C. Chung sp. nov.

CB4D9E10-58F9-52DA-B652-B584F7A3E528

77335215-1

*Rubushowii* auct. non Merr. & Chun, Lu and Yang, Taiwania 25: 123. 1980; Huang and Hu, Taiwania 54(4): 294. 2009.
Previously published illustrations — Lu and Yang (1980: 123, f.2)

#### Materials

- **Type status:** Holotype. **Occurrence:** recordNumber: *S.Y. Lu 22113*; recordedBy: *S.Y. Lu*; occurrenceID: 4449CAE1-85F0-5D42-9E7B-EF1545560C83; **Taxon:** scientificName: *Rubuspuyumaensis*; **Location:** country: Taiwan; county: Taitung; locality: Lichia logging tract; **Event:** year: 1980; habitat: forest edge; **Record Level:** type: specimen; institutionCode: TAIF; collectionCode: TAIF 97606
- **Type status:** Isotype. **Occurrence:** recordNumber: *S.Y. Lu 22113*; recordedBy: *S.Y. Lu*; occurrenceID: 8055762D-8338-54CD-BB95-0CBECC8A2935; **Taxon:** scientificName: *Rubuspuyumaensis*; **Location:** country: Taiwan; county: Taitung; locality: Lichia logging tract; **Event:** year: 1980; habitat: forest edge; **Record Level:** type: specimen; institutionCode: TAIF; collectionCode: TAIF 97607

#### Description

**Figure 1.**
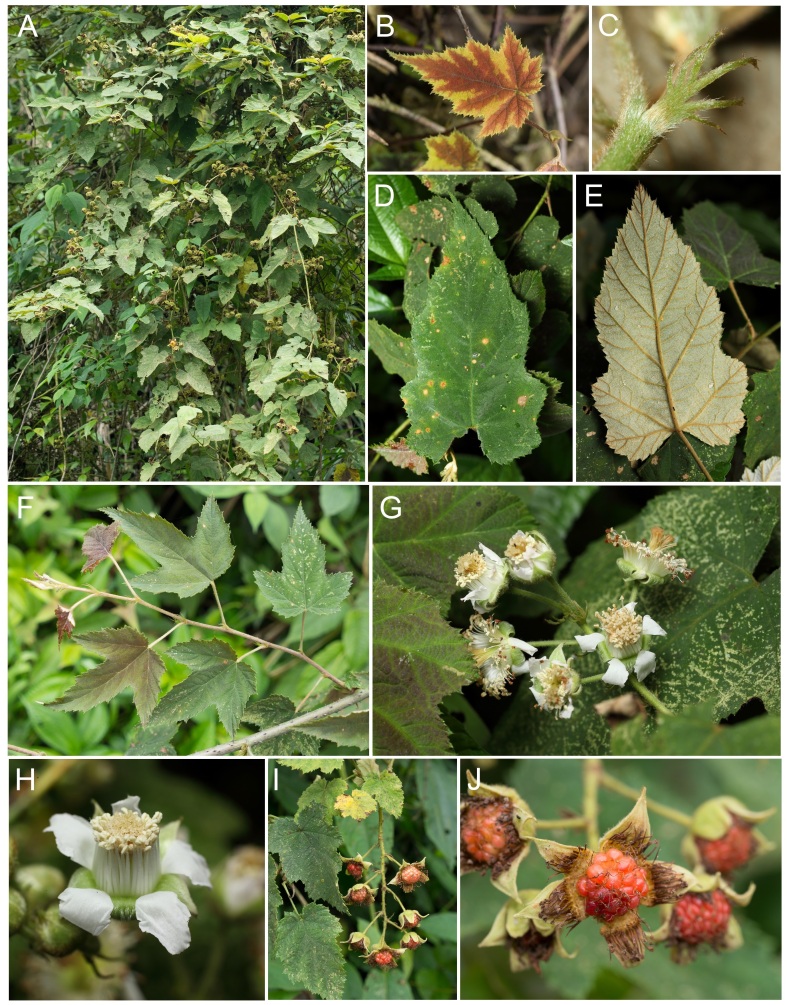
*Rubuspuyumaensis* J.Y. Huang, P.H. Chen & A.C. Chung. **A** fruiting plant in natural habitat; **B** young leaf; **C** stipule; **D** leaf (adaxial surface); **E** leaf (abaxial surface); **F** leaves 3-lobed on vegetative branches; **G** inflorescence; **H** flower; **I** infructescence; **J** fruits. Photographed by J. Y. Huang.

Climbing shrubs (Fig. 1A). Young stems with densely light-tan puberulent and sparsely reverse prickles. Leaves simple, alternate, ovate to long-ovate or tri-lobed, 4–8 × 2–5 cm, veins reticulate, base cordate, 3- or 5-nerved (Fig. 1F), the lower part usually shallowly 2- or 4-lobed, margins serrate, irregularly serrate or slightly undulate, apex acuminate, young leaves usually with purple spots at both surfaces (Fig. 1B), adaxial surfaces sparsely puberulent (Fig. 1D), abaxial surfaces white sparsely puberulent to densely tan puberulent (Fig. 1E); petioles 1–4 cm, white pubescent or densely tan puberulent, with reverse prickles. Stipules deeply and pinnately divided, 6–9 mm long, lobes linear, white or tan puberulent (Fig. 1C). Racemes 4–6 cm long (Fig. 1G), terminal or axillary, florets 3–8, pedicels ca. 1–2 cm long, densely white or tan puberulent; bracts deeply and pinnately divided, 6–8 mm long, lobes long-lanceolate; flowers 1.5 cm in diameter (Fig. 1H), calyx tube cupular, 9–10 mm long, lobes, ovate-lanceolate, 6–7 mm long, apex acuminate, both surfaces white or tan puberulent, reflexed; petals 5, ovate to elliptic, 5–7 × 4–5 mm, base claw-like, apex rounded or obtuse, both surfaces white puberulent; stamens numerous, nearly equal in length with styles; filaments 1–1.2 cm long, pubescent; pistils numerous, ovary ovoid, 2 mm long, puberulent; styles 12–13 mm long, pilose; stigma 1 mm long. Syncarps orange-red to salmon colour in maturity (Fig. 1I, J), globose, 1.5 cm in diameter.

##### Additional specimens examined

TAIWAN: Hualien Co.: Chinshui Farm, *J. Y. Huang 2312* (HAST); Mt. Zhuoxi, *S.W. Chung 14171* (TAIF). Taitung Co.: Taimali, *S.Y. Lu 7211* (TAIF); Lichia logging tract, *J.Y. Huang 2340* (HAST); Yenping Forest Road, *Y.J. Lin 200* (PPI).

#### Diagnosis

*Rubuspuyumaensis* morphologically resembles *R.howii* and *R.refractus*. They can be distinguished by the following characteristics (Table 1). The young leaves of *R.puyumaensis* are often with purple spots and the other two without. There are underdeveloped lobes at the lower part of leaf in *R.howii*, with the other two having 2–4 well-developed lobes. The inflorescences have glandular trichomes and with florets arranged loosely in *R.refractus*; with the other two having no glandular trichomes and florets not loosely arranged. The length of sepal lobes: *R.howii* 6–8 mm, *R.puyumaensis* 6–7 mm, *R.refractus* 7–9 mm. The petal colour is light-tan in *R.howii*, with the other two being white. The filaments are wide, flat in *R.refractus* and narrow, flat in the other two; puberulent in *R.puyumaensis* and glabrous in the other two. The lengths of pistils compared to stamens in *R.howii*, *R.puyumaensis* and *R.refractus* are slightly longer, as long as and conspicuously longer, respectively.

#### Etymology

The new specific epithet commemorates the Puyuma aborigine, a group of indigenous people mainly settled in south-eastern Taiwan and includes the type locality of *Rubuspuyumaensis*.

#### Distribution

Endemic to Taiwan, found in forests edge in the eastern part of the island at medium altitudes 800–1500 m.

##### Vernacular name

Bēi Nán Xuán Gōu Zǐ (Chinese pronunciation); 卑南懸鉤子 (Chinese name).

#### Ecology

Flowering season: March to May; fruiting season: May to July.

#### Conservation

*Rubuspuyumaensis* is currently known to be distributed along three forest roads in the eastern part of Taiwan, including Lijia Forest Roads, Wuluh Forest Roads and Changliang Forest Roads. Its wild population consists of fewer than 1000 individuals at the national level. Therefore, based on the IUCN Red List Categories and Criteria (IUCN 2019), the conservation status assigned to *R.puyumaensis* is "Nationally Vulnerable" [D1+D2].

## Discussion

**Additional specimens examined**:TAIWAN: Hualien Co.: Chinshui Farm, *J. Y. Huang 2312* (HAST); Mt. Zhuoxi, *S.W. Chung 14171* (TAIF). Taitung Co.: Taimali, *S.Y. Lu 7211* (TAIF); Lichia logging tract, *J.Y. Huang 2340* (HAST); Yenping Forest Road, *Y.J. Lin 200* (PPI).

### Recent molecular biology research relevant to the species discussed in this study

In light of morphological and molecular evidence (Huang et al. 2023), the genus *Rubus* consists of 10 subgenera: R.subg.Anoplobatus, R.subg.Batothamnus, R.subg.Chamaerubus, R.subg.Cylactis, R.subg.Dalibarda, R.subg.Idaeobatus, R.subg.Lineati, R.subg.Malachobatus, R.subg.Melanobatus and R.subg.Rubus.

Based on the following morphological characteristics: leaves being simple; stipules broad, usually dissected, free from petioles; and flowers numbering more than 2, we classify *R.puyumaensis*, *R.howii* and *R.refractus* all under R.subg.Malachobatus, as proposed by Huang et al. (2023).

## Supplementary Material

XML Treatment for Rubus puyumaensis

## Figures and Tables

**Table 1.** Diagnostic character differences between *Rubuspuyumaensis* and its morphologically close relatives *R.howii* and *R.refractus*.

Species	*R.puyumaensis*	*R.howii*	*R.refractus*
Young leaves	often with purple spots	without purple spots	without purple spots
Lobes at the lower part of leaf	2–4, well-developed	underdeveloped	2–4, well-developed
Inflorescences	without glandular trichomes; florets not loosely arranged	without glandular trichomes; florets not loosely arranged	with glandular trichomes; florets loosely arranged
Sepal lobes	6–7 mm	6–8 mm	7–9 mm
Petals colour	white	light-tan	white
Filaments	narrow and flat, puberulent	narrow and flat, glabrous	wide and flat, glabrous
Length of pistils	as long as stamens	slightly longer than stamens	conspicuously longer than stamens
